# Tax ubiquitylation and SUMOylation control the dynamic shuttling of Tax and NEMO between Ubc9 nuclear bodies and the centrosome

**DOI:** 10.1186/1742-4690-8-S1-A146

**Published:** 2011-06-06

**Authors:** Youmna Kfoury, Niclas Setterblad, Marwan El-Sabban, Alessia Zamborlini, Zeina Dassouki, Hiba El Hajj, Olivier Hermine, Claudine Pique, Hugues de Thé, Ali Saïb, Ali Bazarbachi

**Affiliations:** 1 Department of Internal Medicine, American University of Beirut, Beirut, Lebanon; 2 Imagery Department of the Institut Universitaire d’Hématologie IFR105, Paris, France; 3 Department of Human Morphology, American University of Beirut, Beirut, Lebanon; 4 UMR 7212 CNRS, U944 Inserm, Laboratoire Associé au Comité de Paris de la Ligue contre le Cancer, Paris, France; 5 CNRS UMR 8603 and Department of Hematology, Necker Hospital, Paris, France; 6 INSERM U1016, CNRS UMR 8104, Université Paris Descartes, Institut Cochin, Department of cell Biology and Host-Pathogens Interactions, Paris, France

## 

The HTLV-I oncoprotein Tax is critical for T cell transformation, acting mainly through NEMO binding and subsequent NF-κB activation. Tax localizes to Tax nuclear bodies and to the centrosome and is subjected to ubiquitylation and SUMOylation that are both necessary for complete transcriptional activation. By using the photoconvertible fluorophore Dendra-2 coupled with live video confocal microscopy, we show for the first time that the same Tax molecule shuttles among Tax nuclear bodies and between these nuclear bodies and the centrosome depending on its post-translational modifications. Ubiquitylation targets Tax to nuclear bodies to which NEMO is recruited and subsequently SUMOylated. We also demonstrate that Tax nuclear bodies contain the SUMOylation machinery including SUMO and the SUMO conjugating enzyme Ubc9, strongly suggesting that these nuclear bodies represent sites of active SUMOylation. Finally, both ubiquitylation and SUMOylation of Tax control NEMO targeting to the centrosome. Altogether, we are proposing a model where both ubiquitylation and SUMOylation of Tax control the shuttling of Tax and NEMO between the cytoplasmic and nuclear compartments.

